# Ginger (*Zingiber officinale* Roscoe), Lemon (*Citrus limon* L.) Juices as Preventive Agents from Chronic Liver Damage Induced by CCl_4_: A Biochemical and Histological Study

**DOI:** 10.3390/antiox11020390

**Published:** 2022-02-15

**Authors:** Oussama Bekkouch, Mohammed Dalli, Mohamed Harnafi, Ilham Touiss, Imane Mokhtari, Soufiane El Assri, Hicham Harnafi, Mohammed Choukri, Seok-Jae Ko, Bonglee Kim, Souliman Amrani

**Affiliations:** 1 Laboratory of Bioresources, Biotechnology, Ethnopharmacology and Health, Faculty of Sciences, Mohammed First University, Oujda 60000, Morocco; harnafimohamed01@gmail.com (M.H.); touissilham@gmail.com (I.T.); imane.mokhtari15@hotmail.com (I.M.); hhicham02@gmail.com (H.H.); amrani137@yahoo.fr (S.A.); 2 Laboratory of Biochemistry, University Hospital Center Mohammed VI, BP 4806, Oujda 60000, Morocco; elassri.bio@gmail.com (S.E.A.); choukrimohammed@hotmail.com (M.C.); 3 Faculty of Medicine and Pharmacy, Mohammed First University, Oujda 60000, Morocco; 4 Department of Gastroenterology, College of Korean Medicine, Kyung Hee University, Seoul 02447, Korea; kokoko119@khu.ac.kr; 5 Department of Pathology, College of Korean Medicine, Kyung Hee University, Seoul 02447, Korea

**Keywords:** *Zingiber officinale*, *Citrus limon*, synergy, antioxidant, hepatoprotection, CCl_4_, liver damage

## Abstract

*Zingiber officinale* Roscoe and *Citrus limon* L. are well known for their multi-use and for their pharmacological effect in the treatment of many illnesses. This study aims to investigate the chemical composition of the ginger and lemon juice extracts and in addition, to evaluate their antioxidant properties and their hepatoprotective effect against the liver damage of Wistar rats induced by the injection of CCl_4_ to treated animals. The obtained effects were completed by a histological study for better confirmation of the registered pharmacological effects. The ginger juice extract was found to be rich in 4-gingerol, 6-gingediol, and 6-gingerol, while the lemon juice extract chemical composition was highlighted by the presence of eriodyctiol, rutin, hesperidin, and isorhamnetin. Concerning the antioxidant activity, the ginger, lemon juice extracts, and their formulation showed an important antioxidant potential using TAC (total antioxidant capacity), an antiradical activity against the radical DPPH• (2,2-diphenyl-1-picrylhydrazil), and a ferric reducing power. Finally, the ginger, lemon, and their formulation at different doses were able to prevent CCl_4_ induced liver damage. Indeed, these different bioactive compounds could be used as alternative agents for the treatment of chronic liver diseases.

## 1. Introduction

The liver is a large gland that performs numerous functions including principally metabolic functions, but also biliary secretion, xenobiotic detoxification, and excretion [1]. However, its integrity is threatened by several toxic external agents (drugs, industrial products, heavy metals, etc.), which can cause cirrhosis, steatosis, necrosis, which are generally grouped under the term hepatotoxicity [2].

In general, acute or chronic exposure to hepatotoxic agents results in a destabilization of the hepatic pro/antioxidant balance because of the excessive production of free radicals. These free radicals can alter any type of molecules or cells, causing very serious damage that may be irreversible in the long term, whether at the structural or functional level [3]. Thus, a good condition of the liver is essential for maintaining the body’s homeostasis, since the liver is a crucial metabolic crossroads in the functioning of the body. Therefore, it is meaningful to protect the liver against toxic attacks and hepatic pathologies that may affect it, by using certain medicinal substances of synthetic or natural origin [4].

Medicinal plants are globally valuable sources of new medicines [5,6]. There are over 1300 medicinal plants used in Europe, 90% of which are collected from wild resources. In the United States, approximately 118 of the top 150 prescription drugs are based on natural sources [7]. In addition, up to 80% of people in developing countries are dependent on herbal medicines for their primary health care, and more than 25% of medicines prescribed in developed countries are derived from wild plant species [8].

In Africa generally and in Morocco especially and as in many developing countries, many patients use the traditional pharmacopeia to face their various health problems [9]. *Zingiber officinale* Roscoe, also called ginger is an aromatic rhizomatous perennial herb, which grows naturally in South Asia [10]. In China, it is an important part of traditional herbal treatment [11]. In addition, ginger is considered one of the benchmark spices in world cuisine generally, and in Morocco in particular [12]. This plant is characterized by a lot of pharmacological effects, such as an hypolipidemic effect [12,13,14], antioxidant activity [14,15,16,17], an antidiabetic effect [18,19], and anticancer activity [15].

The lemon, scientifically named *Citrus limon* L. is a fruit that belongs to the Rutaceae family [20], it is a shrub native to Southeast Asia, cultivated on the Mediterranean coast and in all regions of the world with a semi-tropical climate [21]. Lemon is known to have many beneficial physiological effects like an anti-inflammatory effect [22,23], lipid-lowering activity [24], antioxidative activity [25,26], anticancer, and antimicrobial effects [26].

The present study aimed to evaluate the antioxidant and hepatoprotective effects of *Z. officinale*, *C. limon*, and their formulation. The oxidative stress markers generated by CCl_4_ in hepatocytes were investigated. A histological study of the liver was assessed to confirm the registered effects. Finally, HPLC (high-performance liquid chromatography) was used for better determination of the bioactive compounds responsible for the obtained pharmacological activities.

## 2. Material and Methods

### 2.1. Plant Material

The rhizomes of ginger (*Zingiber officinale* Roscoe) and lemon fruits (*Citrus limon* L.) were purchased from a herbalist in Oujda city. The botanical identification was assessed by a qualified botanist, Professor Fennane Mohammed, from the scientific institute in Rabat. A voucher specimen was deposited at the herbarium of the Faculty of Sciences, Mohamed Premier University (Oujda, Morocco) under the reference number (HUMPOM-352), and (HUMPOM-450), respectively.

### 2.2. Preparation of Zingiber officinale (GJ) and Citrus limon (LJ) Juices

The juice extraction from the two plants was based on crushing the plant material in a blender until the juice was extracted at room temperature (25 °C). Then, the mixture obtained was filtered to obtain the juice. Finally, the collected liquid was put in the oven to be dried.

### 2.3. Quantification of Secondary Metabolites

#### 2.3.1. Total Polyphenol Content

The total polyphenol content of ginger and lemon juices were determined according to [27], where 1 mL of Folin–Ciocalteu reagent (0.2 N) was taken and mixed with 200 µL of each concentration of the ginger juice or lemon juice. After incubating the mixture for about 5 min, an 800 µL of sodium carbonate aqueous solution (7.5% *w*/*v*) was added to all the tubes and left for incubation for about an hour. All measures were carried out in triplicate.

The absorbance was measured at 760 nm against the blank. Gallic acid (GA) was used to generate the calibration curve for further use in the determination of the polyphenol total quantity. The obtained values were expressed by mg GA/g of plant extract.

#### 2.3.2. Total Flavonoids of Ginger and Lemon Juices

The determination of total flavonoid content was based on the formation of a flavonoid aluminum complex with maximum absorption of 430 nm. Amounts of 200 µL of juice at a concentration of 500 µg/mL, 1 mL of distilled water, and 50 µL of sodium nitrate (5%, *w*/*v*) were mixed and incubated for 6 min. Then, 120 µL of aluminum chloride was added (10%, *w*/*v*) to the reaction mixture. A second incubation for 5 min was undertaken and afterwards a basification by adding 400 µL of NaOH (1 M). All measures were carried out in triplicate. The absorbance was measured at 430 nm against the blank. Quercetin (QE) was used to generate the calibration curve. The total flavonoid content was expressed by QE/g of plant extract.

### 2.4. Qualitative and Semi-Quantitative Analysis of Ginger and Lemon Juices

The qualitative analysis of phenolic compounds presents in ginger and lemon extracts was performed using a high-performance liquid chromatography (HPLC) system (Waters Alliance 2695 system, Milford, MA, USA) coupled with a mass spectrometer. Chromatographic separation was performed on a reversed-phase C18 column (250 × 4.6 mm, 5 µm pore size). The mobile phase consisted of solvent A: water–formic acid (90:10 (*v*/*v*)), and solvent B: water, methanol, acetonitrile (40:50:10 (*v*/*v*/*v*)). Phenolic compounds were eluted starting with 88% A and 12% of B for 20 min, followed by 100% of B for 10 min and finally, 88% A and 12% of B for 15 min. The eluent flow rate was equal to 1 mL/min with the injected volume at 20 μL. All the analyses were performed at room temperature. Standard solutions and extracts of ginger and lemon (GJ, and LJ) were dissolved in pure methanol and filtered through a millipore membrane (0.45 µm).

### 2.5. Antioxidant Activity

#### 2.5.1. Antiradical Scavenging Activity against DPPH

The evaluation of the antiradical potential of the ginger and lemon juices was performed according to (Manzocco et al., 1998). From each concentration, 200 µL was pipetted and mixed with 1.8 mL of DPPH (0.5 mM). After 30 min incubation, the absorbance of the reaction mixture was measured at 517 nm. The inhibition percentage of the DPPH free radical was calculated according to the equation:

(1) $$\%\;\mathrm{of}\;\mathrm{Inhibition}\;=\left[\frac{\left(\mathrm{Abs}\;\mathrm{control}-\mathrm{Abs}\;\mathrm{sample}\right)}{\mathrm{Abs}\;\mathrm{control}}\right]\times 100$$

where Abs control is the absorbance of the DPPH and Abs sample is the absorbance of the DPPH plus the extracts tested at 517 nm.

#### 2.5.2. Ferric Reducing Power Assay (FRAP)

The FRAP assay was assessed according to the protocol described by Dehpour et al. [28]. For this, 500 µL of each concentration was mixed with 1.25 mL of phosphate buffer (0.2 M; pH 6.6), and 1.25 mL of K_3_Fe(CN)_6_ (1%, *w*/*v*). Then, the mixture obtained was incubated at 50 °C for about 20 min. After cooling the reaction was stopped by adding 1.25 mL of trichloroacetic acid (10%, *w*/*v*). After that, the mixture was then centrifuged at 1107× *g* for about 10 min. Then, 1.25 mL of the supernatant of each concentration was taken and mixed with 1.25 mL of distilled water and 0.25 mL of ferric chloride solution (FeCl_3_) (0.1%, *w*/*v*). The absorbance of the mixture was measured at 700 nm against the blank. All measurements were carried out in triplicate.

#### 2.5.3. Total Antioxidant Capacity (TAC)

The total antioxidant capacity of the ginger and lemon juices was evaluated using the phosphomolybdate method. This method is based on the reduction of molybdenum (VI), present as an ion molybdate MoO_4_^2−^, to molybdate Mo(V) MoO^2+^, with the formation of a green-colored complex of phosphate/Mo(V) with acid pH. A volume of 300 µL of each concentration was mixed with 3 mL of the reagent solution (0.6 M sulfuric acid, 28 mM of sodium phosphate, and 4 mM of ammonium molybdate). The different tubes were then incubated at 95 °C for about 90 min. After cooling, the absorbance of the different solutions was measured at 695 nm against the blank. The antioxidant total capacity was presented by mg equivalent ascorbic acid/mg of dry weight (mg EAA/g DW). All measures were carried out in triplicate.

### 2.6. Hepatoprotective Activity

Hepatoprotective effect of the ginger and lemon juices against CCl_4_-induced acute liver injury in rats:

#### 2.6.1. Animals

The Wistar rats (250–300 g) used in these experiments were obtained from the animal house of the Faculty of Sciences of Oujda. The rats were maintained in an environmentally controlled room at a temperature of 25 ± 2 °C and a 12-h light/dark cycle. Rats were given free access to food and water. After a 10-day adaptation period, rats were randomly divided into thirteen groups (*n* = 6, each 3 males and 3 females).

#### 2.6.2. Ethical Approval

The study was conducted according to the guidelines of the US National Institutes of Health and approved by the Vice Dean of the Scientific Research of the Faculty of Sciences, University Mohammed the First, Oujda. Indeed, the Vice Dean of the Scientific Research at the Faculty of Sciences, University Mohammed the First of Oujda certified and attested to the total respect of the standards of animal experimentation through a signed and stamped certificate, confirming that all the animal experimentations realized during the present study were carried out in agreement with the internationally accepted Guide for the care and use of laboratory animals.

#### 2.6.3. Experimental Design

The treated groups were injected once a week intraperitoneally by CCl_4_ (1 mL/kg BW) (25% CCl_4_ dissolved in olive oil, *v*/*v*) to induce a liver injury. The animals’ body weight was taken before and after the treatment. The animals were divided into 9 different groups each one containing 6 Wistar rats:

Group 1: control group force-fed with distilled water (10 mL/kg); Group 2: negative control group force-fed with distilled water (10 mL/kg), and injected with CCl_4_ afterward (1 mL/kg); Group 3: treated with ginger juice extract (200 mg/kg) and then injected with CCl_4_; Group 4: treated with ginger juice extract (400 mg/kg) and injected with CCl_4_ thereafter; Group 5: force-fed with lemon juice extract (200 mg/kg) and injected with CCl_4_ afterwards; Group 6: force-fed with lemon juice extract (200 mg/kg) and injected with CCl_4_ afterwards; Group 7: force-fed with ginger juice extract and lemon juice extract (200 mg/kg) and injected with CCl_4_ thereafter; Group 8: force-fed with ginger juice extract and lemon juice extract (400 mg/kg) and injected with CCl_4_ thereafter; Group 9: treated with “Silymarin” (40 mg/kg) and then injected with CCl_4_ afterward.

#### 2.6.4. Histopathological Study

The organs collected after the animal’s sacrifice were weighed and preserved in 10% formalin for 3 days. The method used for the realization of longitudinal sections was the paraffin embedding technique. The histopathological study was performed as follows: in brief, the organs were cleaned with bi-distilled water for 30 min. Then, they were dehydrated by increasing the percentage of ethanol solution (30% for 30 min, 70% for 30 min, 95% for 30 min, and 2 × 100% for 60 min, respectively). After a toluene embedding step (2 × 120 min), the organs were embedded with a paraffin–toluene mixture (1 V/1 V) for 90 min, followed by paraffin (2 × 120 min), the rat livers were then embedded in paraffin before sectioning at 7 μm (Microtome Leitz 1512). The last step consisted in observing the histological sections of the liver with a photonic microscope (MOTIC, Barcelona, Spain) after staining with hematoxylin–eosin. Photographs were taken with a 40× objective ocular system of an Olympus (Tokyo, Japan) light microscope.

### 2.7. Statistical Analysis

All experiments results were performed in triplicate and expressed as means ± SEM. The experimental data were evaluated using GraphPad Prism 9.0.0 (GraphPad Prism Software, Inc.: San Diego, CA, USA) using unpaired Student’s *t*-test for statistical significance between two groups. Then, the analysis of variance (ANOVA) was followed by Tukey’s pairwise comparison test at a 95% confidence interval (*p* < 0.05).

## 3. Results

### 3.1. Extraction and Chemical Composition of Zingiber officinale and Citrus limon

The different dry extracts were obtained by crushing the ginger rhizome and lemon fruits in a blender, the yields obtained were 2.1% and 3.08% for ginger juice and lemon juice respectively. The determination of the total polyphenol content of the different extracts of ginger and lemon juices showed that the different extracts GJ and LJ contained a significant amount of polyphenols. Thus, GJ contains 18.48 ± 1.14 mg gallic acid equivalent/g extract, and LJ contains 25.23 ± 1.54 mg gallic acid equivalent/g extract.

The flavonoid assay revealed a considerable flavonoid content, for the GJ the value recorded was 7.26 ± 2.05 mg eq quercetin/g extract, while for LJ the value obtained was 12.75 ± 2.10 mg eq quercetin/g extract (Table 1).

**Figure 1:**
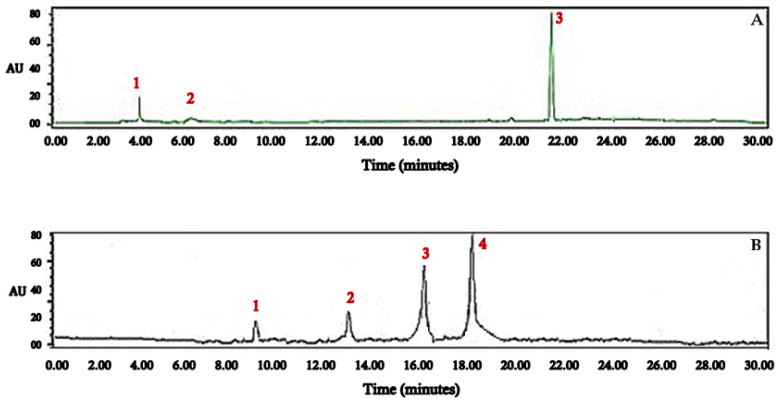
HPLC-MS chromatograms of *Zingiber officinale* (**A**) and *Citrus limon* (**B**) juices.

Concerning the HPLC analysis of the two obtained extracts (GJ, LJ), The results obtained are depicted in Table 2 and Figure 1.

**Figure 2:**
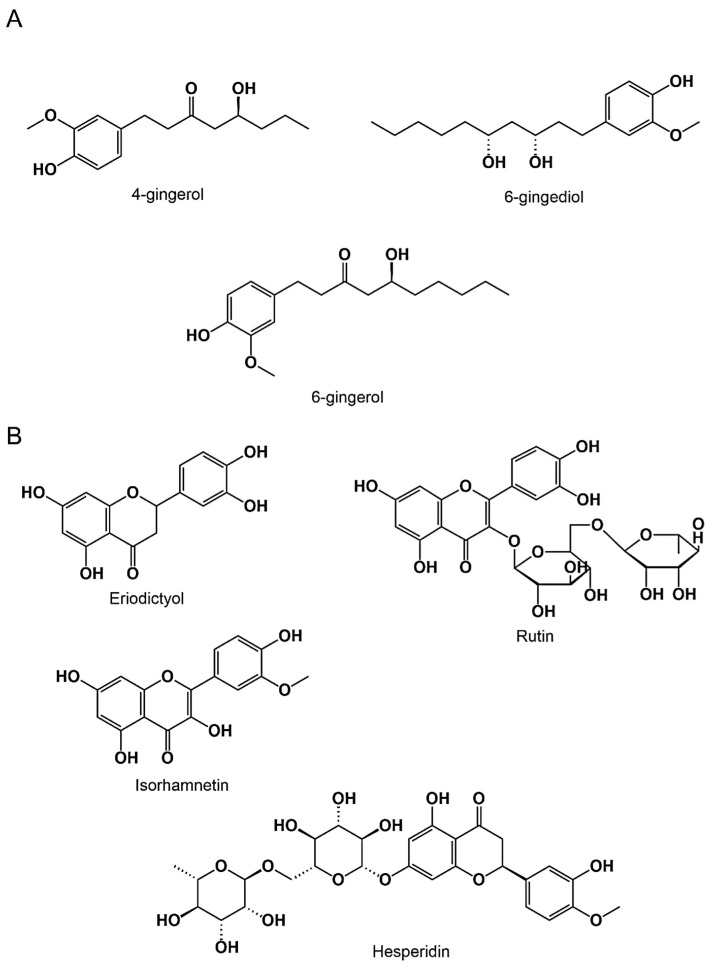
Chemical structure of *Zingiber officinale* (**A**) and *Citrus limon* (**B**) phenolic compounds.

The ginger juice extract was found to be rich in 6-gingerol, 4-gingerol, and 6-gingerol (Figure 1A; Table 2b; Figure 2A), while the chemical analysis of the lemon juice extract revealed the presence of eriodictyol, hesperidin, rutin, and isorhamnetin (Figure 1B; Table 2a; Figure 2B).

### 3.2. Antioxidant Activity

#### 3.2.1. Radical Scavenging Activity

**Figure 3:**
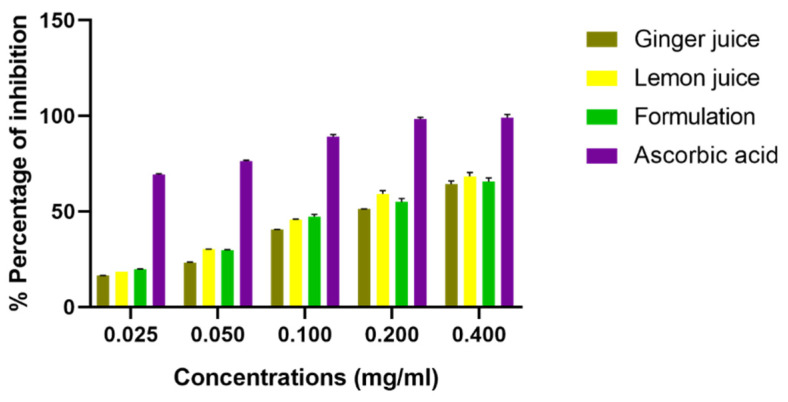
Anti-radical activities of *Zingiber officinale* and *Citrus limon* juices. Values are expressed as “mean ± SEM”; (*n* = 3).

Concerning the antioxidant activity using the free radical DPPH• (Figure 3), the results obtained are drafted in Figure 2. It was noted that the different juice extracts of ginger and lemon showed an important antioxidant activity in a dose-dependent manner.

The LJ extract gave the lowest IC_50_ (16.61 ± 0.78 µg/mL) (*p* < 0.001) compared to the GJ extract which had the highest IC_50_ value equal to 20.35 ± 1.51 µg/mL (*p* < 0.001), while the formulation between ginger (50%) and lemon (50%) juice “F” demonstrated an intermediate effect with an IC_50_ equal to 18.34 ± 0.81 µg/mL (*p* < 0.01). On the counterpart, the ascorbic acid gave the strongest antiradical potential with an IC_50_ of 1.83 ± 0.01 µg/mL (*p* < 0.001).

#### 3.2.2. Ferric Reducing Assay

**Figure 4:**
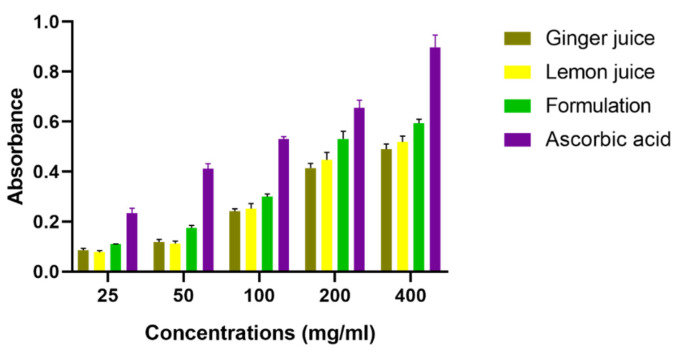
Reducing power activity of *Zingiber officinale* and *Citrus limon* extracts. Values are expressed as “mean ± SEM”; (*n* = 3).

Regarding the ferric reducing power assay, the acquired results are drafted in Figure 4, where it was noticed that both ginger, lemon, and the formulation between them exert an important reducing activity toward ferrous iron (Fe^2+^), indicated by the increase in the level of optical density in the different tested samples. The GJ gave the weakest activity which was indicated by low absorbance values and this among the three studied extracts, followed by the LJ which gave a moderate activity, while the formulation between the two juice extracts (50%/50%) showed a synergic effect that was demonstrated by a high reducing capacity of the Fe^2+^. Finally, the ascorbic acid used as control gave the best reducing activity to reduce the ferrous iron to ferric iron Fe^3+^.

#### 3.2.3. Total Antioxidant Activity

**Figure 5:**
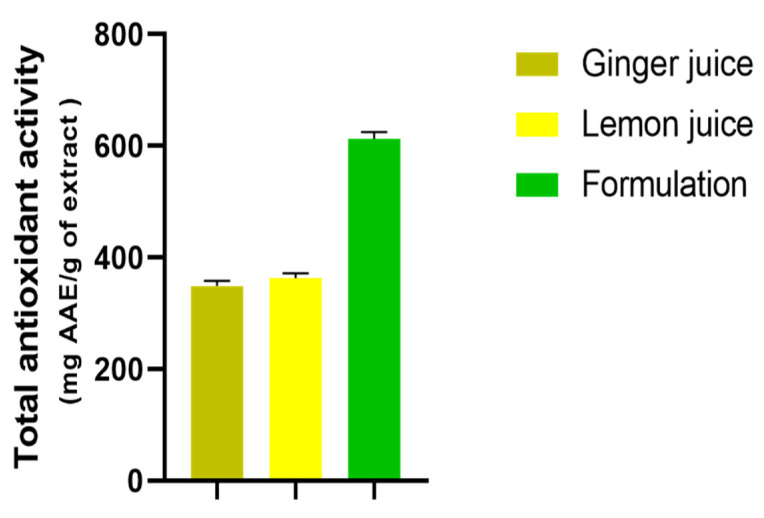
Total antioxidant activity of *Zingiber officinale* and *Citrus limon* extracts. Values are expressed as “mean ± SEM”; (*n* = 3).

The total antioxidant capacity (TAC) was measured according to the phosphomolybdate method. The results of the ginger and lemon extracts in addition to their formulation and total antioxidant activity are displayed in Figure 5 where it is indicated that the different extracts and their formulation exert an important antioxidant activity that was dose-dependent and statistically significant.

The GJ indicated having a weak antioxidant effect among the three extracts, with a value of 348.70 mg AAE/g of extract, followed by LJ, with a value of 363.23 mg AAE/g of extract, while, the formulation F gave the best synergic effect, with a value equal to 612.35 mg AAE/g of extract.

## 4. Hepatoprotective Activity

Table 3 shows that CCl_4_ injection induced a decrease in body weight in rats not treated with extracts. However, the pre-treatment of the rats with both ginger and lemon juices prevented the decrease in body weight, the same finding was observed in liver weight and thus the hepatic index.

### 4.1. Biochemical Analyses

The ginger, lemon juice extracts, and their formulation were evaluated for their hepatoprotective effect against CCl_4_ induced liver injury. The results obtained are tabulated in Table 4. The liver enzymes ALP (alkaline phosphatase), Gamma-GT (gamma-glutamyl transferase), ALAT (alanine transaminase), ASAT (aspartate transaminase), direct bilirubin, and total plasma bilirubin increased significantly (*p* < 0.001) in all animals treated with CCl_4_ compared to the control group. The animals force-fed with GJ, LJ, and their formulation, explicitly prevented in a significant and dose-dependent manner increased levels of the main markers of liver activity (ALP, Gamma-GT, ALAT, ASAT, direct and total bilirubin) (Table 3), compared to the negative control (rats injected with CCl_4_ alone), and compared to the Silymarin drug group, and which gave a very important and statistically significant hepatoprotective effect.

Both GJ and LJ extracts had considerable effects on ALP, while the formulation F gave the best inhibitory effect of ALP from 527.1 ± 10.4 u/L (*p* < 0.001) for the CCl_4_ group, to 295.31 ± 7.08 u/L (*p* < 0.001) and to 222.6 ± 6.98 u/L (*p* < 0.001) at 200 and 400 mg/kg respectively. Concerning the GGT, the GJ gave the best activity among the studied extracts and it was able to block the increase of the GGT from 11.60 ± 0.85 u/L (*p* < 0.001) for the CCl_4_ group, to 6.17 ± 0.55 u/L (*p* < 0.001), and to 3.60 ± 0.15 u/L (*p* < 0.001) at 200 and 400 mg/kg doses, respectively, while, all the tested extracts showed a preventive effect from ALAT and ASAT elevation. In the group treated using the formulation it prevented from the increase of ALAT from 1583.9 ± 14.9 u/L (*p* < 0.001) for the CCl_4_ group, to 490.33 ± 9.63 u/L (*p* < 0.001), and to 363.2 ± 3.65 u/L (*p* < 0.001) at 200 and 400 mg/kg respectively, and ASAT from 5703.8 ± 11.6 u/L (*p* < 0.001) for the CCl_4_ group, to 590.125 ± 10.1 u/L (*p* < 0.001), and to 535.6 ± 7.54 u/L (*p* < 0.001) at 200 and 400 mg/kg respectively. In addition, all extracts prevented the increase of both direct and total bilirubins (Bil D and Bil T), with a greater effect in the formulation F-treated rat groups, which demonstrated an inhibition of direct bilirubin from 1.80 ± 0.05 mg/L (*p* < 0.001) for the CCl_4_ group, to 0.68 ± 0.07 mg/L (*p* < 0.001), and to 0.16 ± 0.01 mg/L (*p* < 0.001) at 200 and 400 mg/kg respectively and total bilirubin from 3.40 ± 0.31 mg/L (*p* < 0.001) for the CCl_4_ group, to 1.15 ± 0.07 mg/L (*p* < 0.001), and to 0.72 ± 0.03 mg/L (*p* < 0.001) at 200 and 400 mg/kg respectively. Concerning the control group treated with Silymarin, a significant decrease was shown with the group treated with a dose of 25 mg/kg compared to CCl_4_-treated rats alone in ALP levels by −63.09% (*p* < 0.001), ASAT by −88.07% (*p* < 0.001) and ALAT by −96.39% (*p* < 0.001), while it had no inhibitory effect on plasma levels of Gamma-GT, direct and total bilirubin (Table 2).

Finally, it was demonstrated that ginger and lemon juices and their formulation possess significant hepato-protective effects more or less significantly and in a dose-dependent manner, which allows us to say that the juice extracts of ginger and lemon are very efficient for liver pathologies.

### 4.2. Histopathological Study

**Figure 6:**
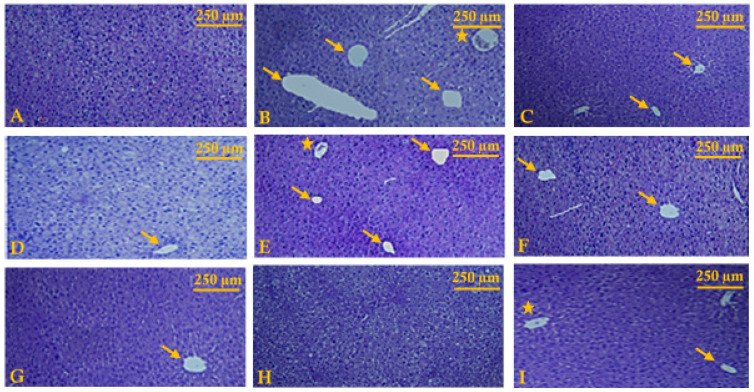
Effect of *Zingiber officinale* and *Citrus limon* extracts on CCl_4_-induced hepatotoxicity. (**A**) micrograph of the livers of control rats showing normal histological liver structure. (**B**) Rats that received CCl_4_ (1 mg/kg) showed drastic damage to hepatocytes reflected by a fatty change in hepatocytes leading to the appearance of steatosis (**Arrows**), and the appearance of congested blood vessels (**Stars**) as well as areas of liver necrosis. (**C**–**I**) rats that received CCl_4_ + GJ (200 mg/kg), CCl_4_ + GJ (400 mg/kg), CCl_4_ + LJ (200 mg/kg), CCl_4_ + LJ (400 mg/kg), CCl_4_ + F (200 mg/kg), CCl_4_ + F (400 mg/kg), and CCl_4_ + Silymarin (40 mg/kg) respectively, showed a quasi-regeneration of the liver tissue giving a comparable tissue appearance to that of the control group and presenting a normal histological liver structure, a minor effect of steatosis formation as well as a decrease in the number of congested blood vessels in a dose-dependent manner.

The biochemical effects obtained from this study were accompanied and confirmed by a histological study of different rats’ livers (Figure 6). Microscopic observation in slides of liver tissue from the different groups of rats shows that administration of the hepatotoxic agent (CCl_4_) induces a change in the general morphology of the liver tissue compared with the control, which has an ordinary structure.

These alterations vary from simple congestion of the vessels to necrosis and hepatocellular degeneration (structural and functional loss). However, treatment with the various doses of extracts of ginger and lemon juices, and their formulation was effectively able to improve these alterations, since the hepatic tissue aspect was almost regenerated with a normal hepatic aspect, following regeneration of the plasma membrane and repair of the lobular architecture.

## 5. Discussion

Oxidative stress is mainly related to reactive oxygen species production which is defined as oxygen radicals and chemically hyper-reactive non-radical oxygen derivatives. These formed radicals are disactivated through the intrinsic antioxidant system which plays a crucial role in detoxifying the body from the production of radicals that occurs during the biochemical process [29]. Despite the presence of enormous intrinsic antioxidant mechanisms, oxidative damage remains an inevitable result. Recently, it was demonstrated that oxidative stress is associated with a wide range of degenerative processes, diseases, and syndromes [30]. In addition, oxidative stress was found to be related to different pathologies such as cardiovascular disease, respiratory disorder, chronic kidney disease, neurodegenerative, and cancer disease [31].

Regarding the different chemical profiles identified using high-performance liquid chromatography, and the antioxidant activity, the hepatoprotective effect could be attributed to the different bioactive compounds present in each extract such as phenolic compounds and flavonoids.

The LJ extract showed a polyphenol and flavonoid content equivalent to 25.23 ± 1.54 mg GA/g extract, and 12.75 ± 3.08 mg QE/g extract, respectively.

This same extract was found to be rich in eriodictyol, rutin, hesperidin, and isorhamnetin, while, the GJ extract showed values corresponding to 18.48 ± 1.14 mg GA/g extract and 7.26 ± 2.05 mg QE/g extract for the polyphenol and flavonoid content, respectively. The chemical analysis showed the existence of 4-gingerol, 6-gingediol, and 6-gingerol. Our results were quite similar to those obtained in the study realized by Tiencheu et al., where it was reported that both ginger, lemon, and their formulation contain a considerable amount of phenolic compounds and flavonoids [32].

Concerning the antioxidation potentials of ginger and lemon juices, the antioxidant activity was first performed using the free radical DPPH•, the ferric reducing power assay, and the total antioxidant activity. The results obtained indicate that the GJ and LJ and their formulation at different concentrations were endowed with a great antiradical scavenging activity with an IC_50_ value of 20.35 ± 1.50 µg/mL and 16.61 ± 0.78 µg/mL for the GJ and LJ respectively, which was found to be correlated positively with the total polyphenol content of the tested extracts. Furthermore, ginger and lemon juice extracts and their formulation demonstrated a great reduction potential of Fe^2+^, indicated by the increase of the optical density, and IC_50_ equivalent to 403.21 ± 5.75 µg/mL, 391.17 ± 3.64 µg/mL, and 188 ± 2.11 µg/mL attributed to GJ, LJ and the formulation F respectively. In addition, a synergic effect was observed when using the formulation F of the ginger and the lemon converted by an IC_50_ = 188 ± 2.11 µg/mL. Similarly, it was found that ginger juice possesses great antiradical and ferric reducing power activities [33], while it was found that citrus juice is endowed with an important antioxidant activity [33,34,35]. In addition, it is noteworthy to mention that the lemon juice extract antioxidant activity was similar to the results presented by Makni et al., where it was shown that the ferric reducing power of the lemon extract was equivalent to 90 µg/mL [36].

Regarding the total antioxidant capacity, the GJ, LJ, and their formulation exerted an important antioxidant potential in a dose-dependent manner. Among all the tested extracts the formulation between the two juices showed an effective synergy compared to each extract tested separately.

Indeed, the study performed on the aqueous extract showed very effective activity when tested using the molybdenum test [37], while the studies investigating the lemon ethanolic extracts indicated an important antioxidant potential [38]. In parallel, it has been shown that lemon peel and fruits demonstrate a high total antioxidant potential [39,40].

The gingerols, one of the major bioactive compounds in ginger have shown significant antioxidant properties [41,42]. The antioxidant activities of lemon juice extract are inevitably due to the different bioactive compounds identified using HPLC. Several studies have investigated these different molecules separately such as the case of eriodictyol where the high antioxidative potential of the molecule was mentioned [43], while rutin has registered an antioxidant activity equivalent to 2.03 ± 0.01 mmol Trolox [44]. In the same context, several studies have demonstrated the antioxidant capacity of the isorhamnetin and hesperidin [45,46,47,48].

Concerning the hepatoprotective activity of the LJ, GJ, and their formulation, this was evaluated using the CCl_4_ model. The hepatoxic effect is induced by the administration of CCl_4_ which is reduced on the cytochrome P450 2E1 into two reactive and highly unstable free radicals, the trichloromethyl radical (CCl_3_•) and the trichloromethyl peroxyl radical (Cl_3_COO•) [49]. The formation of these two unstable free radicals is the origin of cell damage by inducing a membrane lipid peroxidation, associated with the release of cytosolic and endoplasmic enzymes, which indicate damage to liver structure and function [50]. The damage induced at the liver level is traduced by the elevation of the different liver markers such as ASAT, ALAT, ALP, and GGT.

For a better understanding of the hepatoprotective effect of the tested extracts the liver enzymes markers were measured. The results of ginger, lemon, and the formulation effect on CCl_4_-treated rats are tabulated in Table 2. A significant increase of the liver enzyme markers was observed in the CCl_4_-treated group (*p* < 0.001) in comparison with the control group that received distilled water, while there was a significant decrease of the different measured markers in the positive control group after treatment with Silymarin (25 mg/kg).

It was found that the hepatotoxic effect of the CCl_4_ could be alleviated after the administration of the ginger, lemon juices, and also their 50%/50% formulation. Our results were comparable to those obtained by Oke et al., Hasan et al., Abdel-Azeem et al., who demonstrated that ginger extract plays an important role in the prevention of CCl_4_ induced liver toxicity [51,52,53]. Additionally, in the studies performed by Jaiswal et al., and Bhavsar et al., it was shown that the lemon extracts were able to protect the liver from the harmful effects of CCl_4_ which was in accordance with the results obtained in our study.

The capacity of the tested extract to restore the liver enzyme markers to normal could be attributed to the different bioactive compounds identified using HPLC. Several previous studies have demonstrated the hepatoprotective effect of different major compounds found in ginger and lemon extract. The lemon biomolecules such as isorhamnetin, rutin, eriodictyol, and hesperidin were all reported to possess a preventive effect against liver damage induced by CCl_4_ [54,55,56,57]. On the other hand, It was shown that the protective effect of ginger is due to its bioactive compounds [58]. In addition, the treatment with 6-gingerol had a significant hepatoprotective effect from the undesirable effect caused by acetaminophen [59].

Finally, at the histopathological level, the administration of a hepatotoxic agent, whatever its nature, its dose, or its route of administration, for example CCl_4,_ causes modification of the membrane permeability followed by tissue damage: cell necrosis, hepatic cell lysis, damage to the lysis of liver cells, damage to the bile ducts and/or loss of the functional integrity of the liver tissue architecture [60].

The main consequence of liver damage is increased serum levels of liver enzymes (G-GT, ALP, ALT, and AST) [61]. This reflects the presence of a rupture of the hepatic plasma membrane because these enzymes are primarily intracellular. AST, for example, is found in hepatocytes as two isoenzymes, one cytoplasmic, the other mitochondrial, and therefore, the presence of this enzyme in the extracellular medium signals the presence of damage in the liver cell [62].

On the other hand, exposure to hepatotoxic agents, which leads to liver parenchymal injury, also leads to an elevation of plasma bilirubin concentration [63]. This can be explained by damage to the bile ducts or affection of the erythrocyte membrane by reactive species thus leading to hemolysis, and finally, the elevation of bilirubin levels [64].

In the present study, the ginger, lemon, and their formulation had a high antioxidant potential, while the administration of the two juice extracts and the formulation showed a great capacity to restore the different liver enzymes to normal against the harmful effects induced by the injection of the CCl_4_. These different obtained effects could be attributable to the different bioactive compounds identified using HPLC.

## 6. Conclusions

The finding of the present study revealed that ginger and lemon juices and their formulation are endowed with a significant antioxidant potential which was evaluated using the free radical DPPH•, the ferric reducing power assay (FRAP), and the total antioxidant capacity (TAC). In addition, it was found that the different tested extracts including the formulation have a significant preventive effect against the liver damage induced by CCl_4_. These different obtained effects are mainly attributed to the various bioactive compounds identified using HPLC such as gingerol, gingediol, eriodyctiol, rutin, hesperidin, and isorhamnetin. Further studies need to be undertaken for further use of these plants as alternative agents for the prevention of liver chronic diseases.

## Figures and Tables

**Table 1:** The polyphenol and flavonoid contents of *Zingiber officinale* and *Citrus limon* juices.

	GJ (mg GAE/g Extract)	LJ (mg QE/g Extract)
Total polyphenol content	18.48 ± 1.14	25.23 ± 1.54
Total flavonoid content	7.26 ± 2.05	12.75 ± 2.10

**Table 2:** Chemical composition of *Zingiber officinale* (**a**) and *Citrus limon* (**b**) juices.

(a) Peak Number	Compound	Retention Time (min)	% of Area
1	4-gingerol	3.97	0.81
2	6-gingediol	6.41	0.19
3	6-gingerol	21.60	15.22
**(b) Peak Number**	**Compound**	**Retention Time (min)**	**% of Area**
1	eriodictyol	9.17	3.12
2	rutin	13.25	5.69
3	hesperidin	16.31	13.88
4	isorhamnetin	18.23	18.43

**Table 3:** Effect of *Zingiber officinale* and *Citrus limon* juices on body weight, liver weight, and the liver index.

	Rats Weight (g)	Liver Weight (g)	Liver Index (%)
Control	253.46 ± 3.85	6.85 ± 0.27	2.70
CCl_4_	194.05 ± 4.21 ^a^	10.24 ± 0.31 ^a^	5.28 ^a^
GJ 200	208.91 ± 3.65 ^c^	9.31 ± 0.22 ^c^	4.31 ^c^
GJ 400	223.87 ± 5.72 ^a^	8.34 ± 0.40 ^b^	3.73 ^b^
LJ 200	210.66 ± 2.33 ^c^	8.89 ± 0.28 ^b^	4.22 ^c^
LJ 400	225.90 ± 4.91 ^a^	7.95 ± 0.71 ^a^	3.52 ^b^
F 200	219.71 ± 2.27 ^b^	8.10 ± 0.35 ^a^	3.69 ^b^
F 400	238.12 ± 3.98 ^a^	7.13 ± 0.86 ^a^	2.99 ^a^
Silymarin	225.36 ± 5.54 ^a^	7.77 ± 0.43 ^a^	3.45 ^b^

GJ: ginger juice; LJ: lemon juice; F: formulation; Values are expressed as “mean ± SEM”; CCl_4_ group was compared with the control group; ginger and lemon juices, and silymarin treated groups were compared to the CCl_4_ group; a: *p* < 0.001; b: *p* < 0.01; c: *p* < 0.05; NS: not significant.

**Table 4:** Effects *of Zingiber officinale* and *Citrus limon* extracts on liver markers in CCl_4_-intoxicated rats.

	ALP (u/L)	G-GT (u/L)	ALAT (u/L)	ASAT (u/L)	D-Bil (mg/L)	T-Bil (mg/L)
Group 1	183.8 ± 5.37	3.20 ± 0.12	81.34 ± 1.75	90.10 ± 3.51	0.10 ± 0.01	1.00 ± 0.05
Group 2	527.1 ± 10.4 ^a^	11.60 ± 0.85 ^a^	1583.9 ± 14.9 ^a^	5703.8 ± 11.6 ^a^	1.80 ± 0.05 ^a^	3.40 ± 0.31 ^a^
Group 3	382.70 ± 6.14 ^a^	6.17 ± 0.55 ^a^	1469.3 ± 13.8 ^c^	1708.03 ± 15.5 ^a^	0.47 ± 0.05 ^a^	1.60 ± 0.03 ^a^
Group 4	257.21 ± 5.42 ^a^	3.60 ± 0.15 ^a^	789.1 ± 10.3 ^a^	573.3 ± 11.6 ^a^	0.20 ± 0.01 ^a^	0.90 ± 0.02 ^a^
Group 5	397.00 ± 8.23 ^a^	10.58 ± 1.02 ^NS^	1343.75 ± 12.7 ^c^	903.4 ± 12.3 ^a^	0.73 ± 0.04 ^a^	1.48 ± 0.07 ^a^
Group 6	295.15 ± 4.87 ^a^	7.05 ± 0.80 ^b^	694.03 ± 9.79 ^a^	713.57 ± 9.94 ^a^	0.27 ± 0.01 ^a^	0.97 ± 0.03 ^a^
Group 7	295.31 ± 7.08 ^a^	9.10 ± 0.79 ^c^	490.33 ± 9.63 ^a^	590.125 ± 10.1 ^a^	0.68 ± 0.07 ^a^	1.15 ± 0.07 ^a^
Group 8	222.6 ± 6.98 ^a^	7.90 ± 0.46 ^b^	363.2 ± 3.65 ^a^	535.6 ± 7.54 ^a^	0.16 ± 0.01 ^a^	0.72 ± 0.03 ^a^
Group 9	194.55 ± 5.72 ^a^	37.33 ± 1.81 ^a^	188.9 ± 6.54 ^a^	205.4 ± 8.12 ^a^	4.67 ± 0.73 ^a^	3.40 ± 0.25 ^a^

Values are expressed as “mean ± SEM” of six rats in each batch; Group 1: untreated control group force-fed with distilled water; Group 2: Untreated group; CCl_4_ + Group 3: Ginger juice extract-treated group at the dose of 200 mg/kg; Group 4: Ginger juice extract-treated group at the dose of 400 mg/kg; Group 5: Lemon juice extract-treated group at 200 mg/kg; Group 6: Lemon juice extract-treated group at 400 mg/kg; Group 7: Ginger juice and lemon juice extract-treated group at 200 mg/kg Group 8: Ginger juice and lemon juice extract-treated group at 400 mg/kg; group 9: Silymarin treated group at 40 mg/kg. Group 3, 4, 5, 6, 7, 8, and 9 groups were compared with Group 2. a: *p* < 0.001; b: *p* < 0.01; c: *p* < 0.05; NS: not significant.

## Data Availability

Data is contained within the article.
